# Supplementary material for: Polypharmacology-based kinome screen identifies new regulators of KSHV reactivation
Source: bioRxiv. 2023 Feb 1:2023.02.01.526589. Preprint. [Version 1] doi: 10.1101/2023.02.01.526589 (PMC9915688; doi:10.1101/2023.02.01.526589)
Supplement: Supplement 1 [file NIHPP2023.02.01.526589v1-supplement-1.pdf]

bioRxiv preprint doi: https://doi.org/10.1101/2023.02.01.526589; this version posted February 1, 2023. The copyright holder for this preprint (which was not certified by peer review) is the author/funder, who has granted bioRxiv a license to display the preprint in perpetuity. It is made available under aCC-BY 4.0 International license.

Fig S1: Expression of LANA and the lytic replication indicator SBP-ΔLNGFR from KSHV^{LRI}. Uninfected iSLK cells or KSHV BAC16 or KSHV^{LRI} latently infected iSLK cells were **(A)** lysed and subjected to LANA immunoblotting or **(B)** analyzed by immunofluorescence for LANA puncta representing individual KSHV episomes. **(C)** KSHV BAC16 or KSHV^{LRI} latently infected iSLK cells were treated with 1 μg/ml DOX plus 1 mM NaB and incubated for 3-days before harvesting cells for immunoblot analysis of SBP-ΔLNGFR protein levels or **(D)** fixed and incubated with streptavidin-680 for imaging of SBP-ΔLNGFR on the plasma membrane of un-permeabilized cells.

bioRxiv preprint doi: https://doi.org/10.1101/2023.02.01.526589; this version posted February 1, 2023. The copyright holder for this preprint (which was not certified by peer review) is the author/funder, who has granted bioRxiv a license to display the preprint in perpetuity. It is made available under aCC-BY 4.0 International license.

Fig S2: Polypharmacology-based kinome screen in the absence of KSHV lytic inducing agents. KSHV^{LRI} reactivation phenotypes were obtained from 20 of the 29 pre-selected kinase inhibitors that did not cause cellular toxicity. KSHV reactivation for control (black bar and dotted black line) and kinase inhibitor treatment (red bars) were calculated as a percent of DOX plus NaB treated cells set to 100 from data in Fig 2C. In this graph, 1.0 represents ~0.2% total cells and the dotted line represents spontaneous reactivation, ~0.02% total cells.

Fig S3: Kinase knockdown efficiencies for kinases validated from screen. Knockdown efficiencies for siRNAs targeting specific cellular kinases were evaluated in KSHV^{LRI} latently infected iSLK cells using RT-qPCR from total RNA harvested at 3-days post transfection with siRNAs.

Fig S4: Knockdown efficiencies and specificity for ERBB and MKNK family members. (A) Knockdown efficiency of ERBB2 targeting siRNA was evaluated by immunoblot for ERBB2 protein at 2 days following siRNA transfection of KSHV^{LRI} latently infected iSLK cells. Knockdown specificity for siRNAs targeting **(B)** ERBB or **(C)** MKNK family kinases were evaluated in KSHV^{LRI} latently infected iSLK cells using RT-qPCR from total RNA harvested at 3-days post transfection with siRNAs.

bioRxiv preprint doi: https://doi.org/10.1101/2023.02.01.526589; this version posted February 1, 2023. The copyright holder for this preprint (which was not certified by peer review) is the author/funder, who has granted bioRxiv a license to display the preprint in perpetuity. It is made available under aCC-BY 4.0 International license.

Fig S5: Effects of ERBB2 and reactivation on phosphorylation of downstream signaling factors. KSHV^{LRI} latently infected iSLK cells were transfected with siRNA control or siRNAs targeting ERBB2 and then 3-days later untreated or treated with DOX plus NaB for 24h. Cells were harvested, and protein lysates were analyzed using a RPPA for phosphorylation of **(A)** plasma membrane receptor PDGFRβ at Tyr^{1009} and **(B)** signaling intermediates (pan) PKC at Ser^{660}. **(C)** Cell viability (grey bars) and KSHV reactivation (red bars) were measured for KSHV^{LRI} latently infected iSLK cells transfected with siRNAs targeting individual JAK family kinases and 3-days later uninduced or treated with DOX alone for 72h. Control siRNA transfected cells treated with DOX (dotted black lines) were set to 100 and data for each condition was calculated as a percent of this control. Kinase knockdown efficiencies at 3-days following siRNA transfection were determined before addition of lytic inducing drugs and graphed in Fig S6. For each knockdown, the efficiencies were averaged and listed below the corresponding kinase target as % KD. Identical to (A and B), quantification of phosphorylated **(D)** MARKS at Ser$^{152/156}$, **(E)** S6 at Ser$^{240/244}$, **(F)** NFκB P65 at Ser^{536}, and **(G)** total protein for β-catenin was analyzed. P-values * ≤ 0.05 and ** ≤ 0.01.

Fig S6: Knockdown efficiencies and specificity for JAK family members. (A) Knockdown specificity for siRNAs targeting JAK1, JAK2, JAK3 and TYK2 were evaluated using RT-qPCR from total RNA harvested at 3-days post transfection with siRNAs. Relative mRNA levels for JAK3 were below the level of detection for these samples. **(B)** HeLa cells were transfected with control or JAK3 targeting siRNA alone or in combination with a JAK3 expressing plasmid. Three days post transfection cells were harvested, and lysates were subjected to α-JAK3 and α-Actin immunoblotting.

bioRxiv preprint doi: https://doi.org/10.1101/2023.02.01.526589; this version posted February 1, 2023. The copyright holder for this preprint (which was not certified by peer review) is the author/funder, who has granted bioRxiv a license to display the preprint in perpetuity. It is made available under aCC-BY 4.0 International license.

Supporting Information (SI) Captions

S1 Table. Kinase inhibitors list.

S2 Table. DOX plus NaB condition KiR screen final predicted kinase inhibitors.

S3 Table. DOX plus NaB condition KiR screen final predicted kinases.

S4 Table. Kinase expression data from KSHV BAC16 infected iSLK cells.

The gene expression data normalized as fragments per kilobase of exon per million mapped fragments (FPKM) were taken from the published RNA-seq dataset GSE157275 [34] for the kinases predicted from the kinome screen under lytic induction and the additional ERBB and MKNK family members. These data were organized into a table showing the normalized kinase expression data from BAC16 latently infected iSLK cells and lytically induced cells at 48h post treatment with 50 μg/ml DOX plus 1.2 mM NaB.

S5 Table. Primers.

S6 Table. Dharmacon siRNA target and ID.

S7 Table. Antibodies.

Figure S1

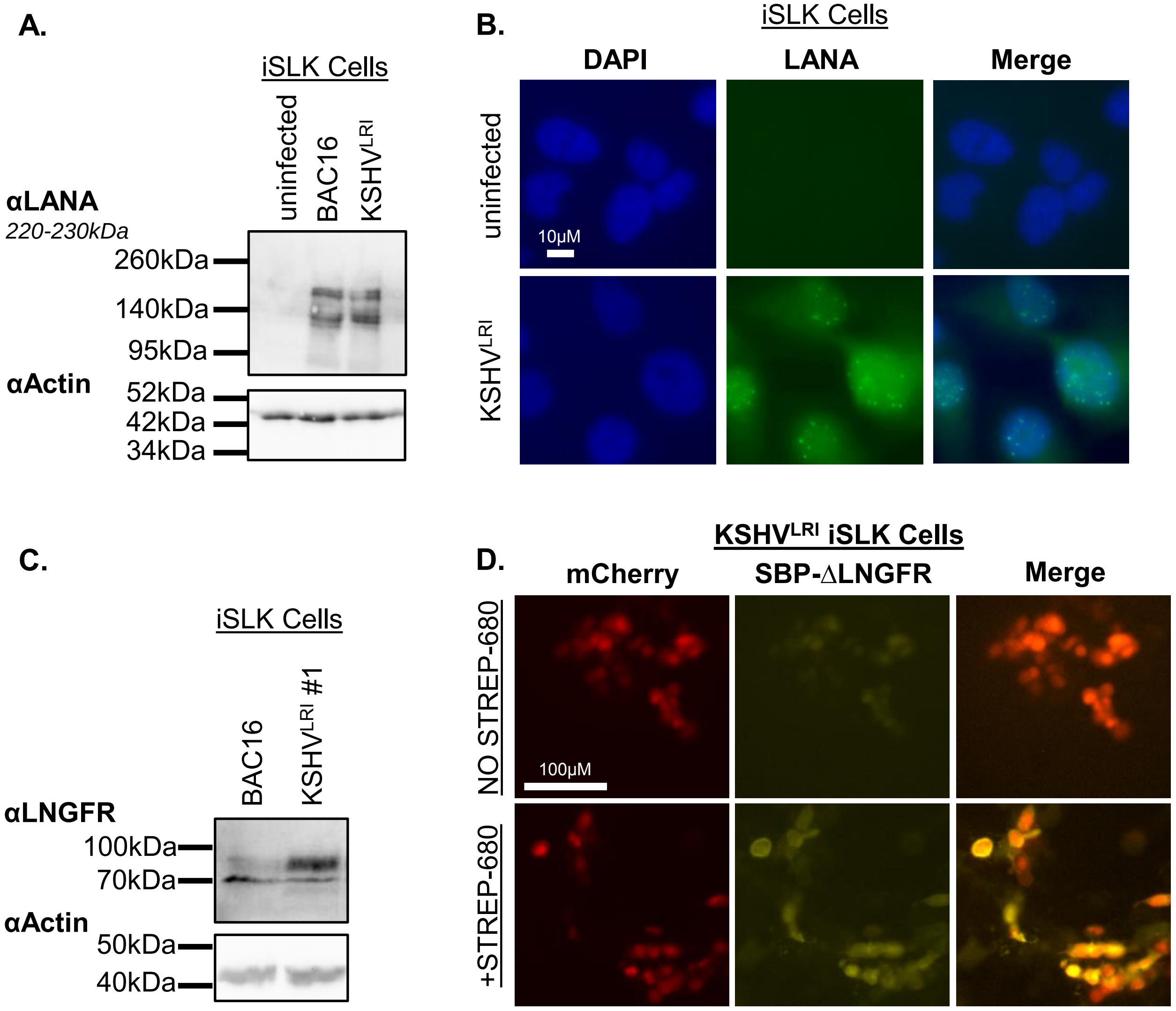
A.
iSLK Cells
uninfected
BAC16
KSHV^LRI
αLANA
220-230kDa
260kDa
140kDa
95kDa
αActin
52kDa
42kDa
34kDa
B.
iSLK Cells
DAPI
LANA
Merge
uninfected
KSHV^LRI
10μM
C.
iSLK Cells
BAC16
KSHV^LRI #1
αLNGFR
100kDa
70kDa
αActin
50kDa
40kDa
D.
KSHV^LRI iSLK Cells
mCherry
SBP-ΔLNGFR
Merge
NO STREP-680
+STREP-680
100μM

Figure S2

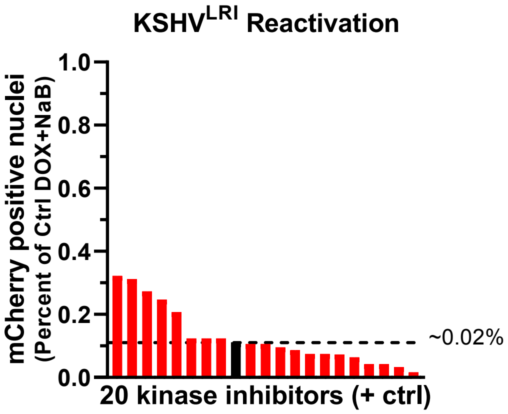

KSHV^LRI Reactivation
mCherry positive nuclei
(Percent of Ctrl DOX+NaB)
1.0
0.8
0.6
0.4
0.2
0.0
~0.02%
20 kinase inhibitors (+ ctrl)

Figure S3

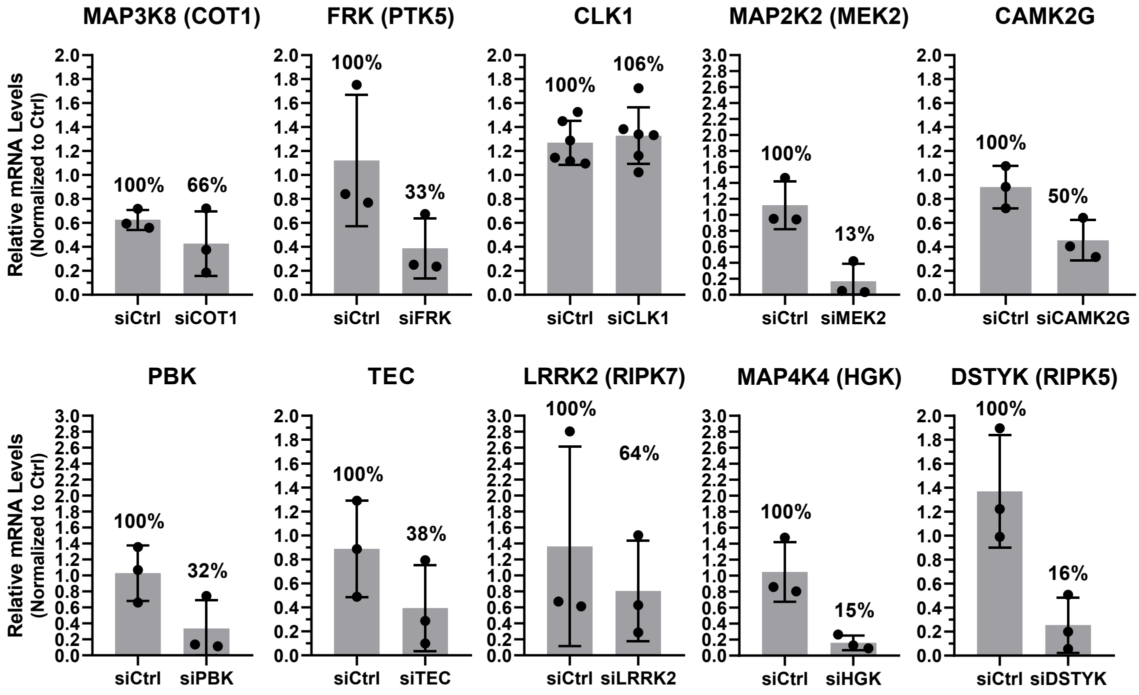
MAP3K8 (COT1)
FRK (PTK5)
CLK1
MAP2K2 (MEK2)
CAMK2G
Relative mRNA Levels (Normalized to Ctrl)
100%
66%
33%
106%
13%
50%
siCtrl
siCOT1
siFRK
siCLK1
siMEK2
siCAMK2G
PBK
TEC
LRRK2 (RIPK7)
MAP4K4 (HGK)
DSTYK (RIPK5)
32%
38%
64%
15%
16%
siPBK
siTEC
siLRRK2
siHGK
siDSTYK

Figure S4

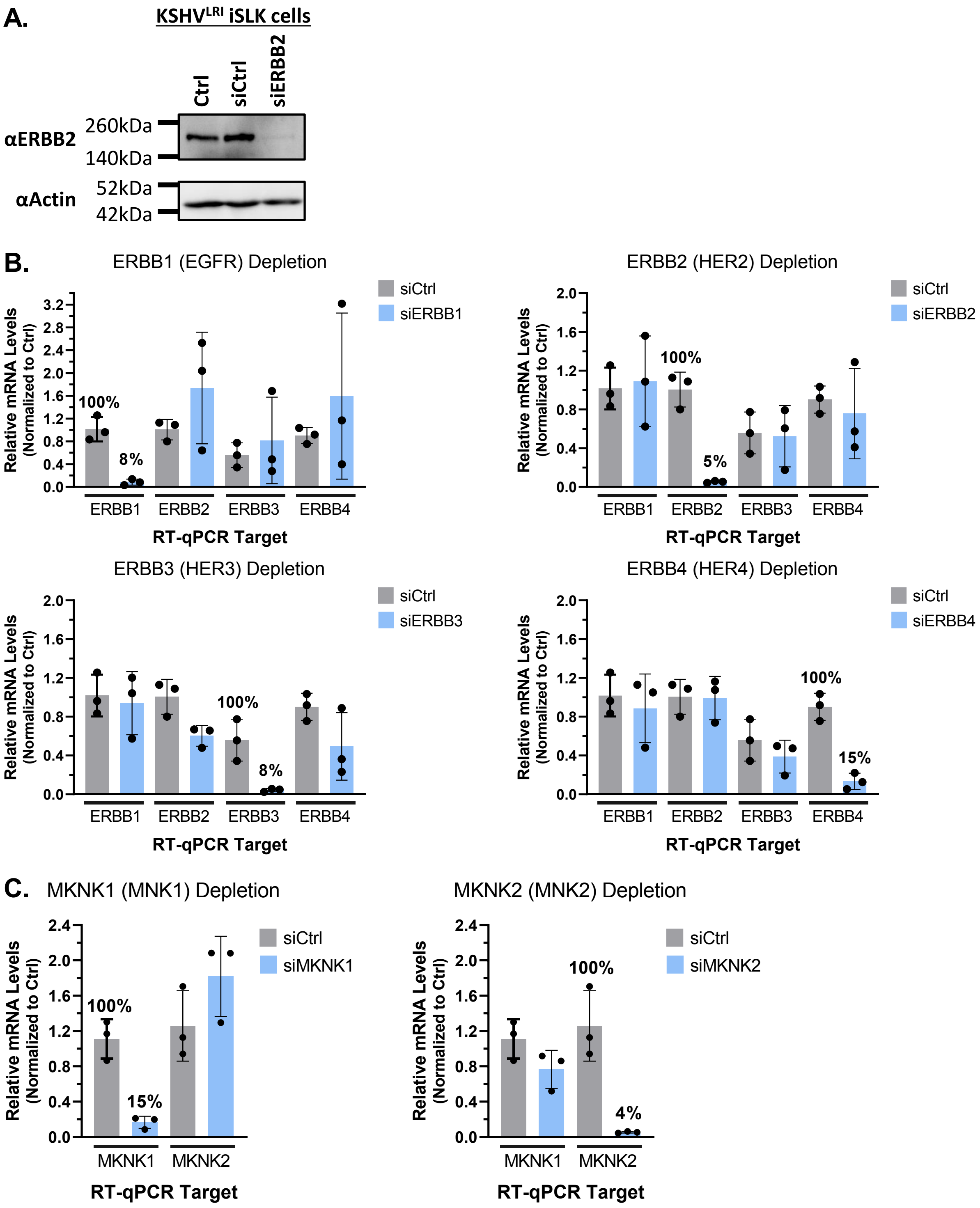
A.
KSHV[LRI] iSLK cells
Ctrl
siCtrl
siERBB2
αERBB2
260kDa
140kDa
αActin
52kDa
42kDa
B.
ERBB1 (EGFR) Depletion
siCtrl
siERBB1
Relative mRNA Levels (Normalized to Ctrl)
100%
8%
ERBB1
ERBB2
ERBB3
ERBB4
RT-qPCR Target
ERBB2 (HER2) Depletion
siCtrl
siERBB2
100%
5%
ERBB3 (HER3) Depletion
siCtrl
siERBB3
100%
8%
ERBB4 (HER4) Depletion
siCtrl
siERBB4
100%
15%
C.
MKNK1 (MNK1) Depletion
siCtrl
siMKNK1
100%
15%
MKNK1
MKNK2
RT-qPCR Target
MKNK2 (MNK2) Depletion
siCtrl
siMKNK2
100%
4%

Figure S5

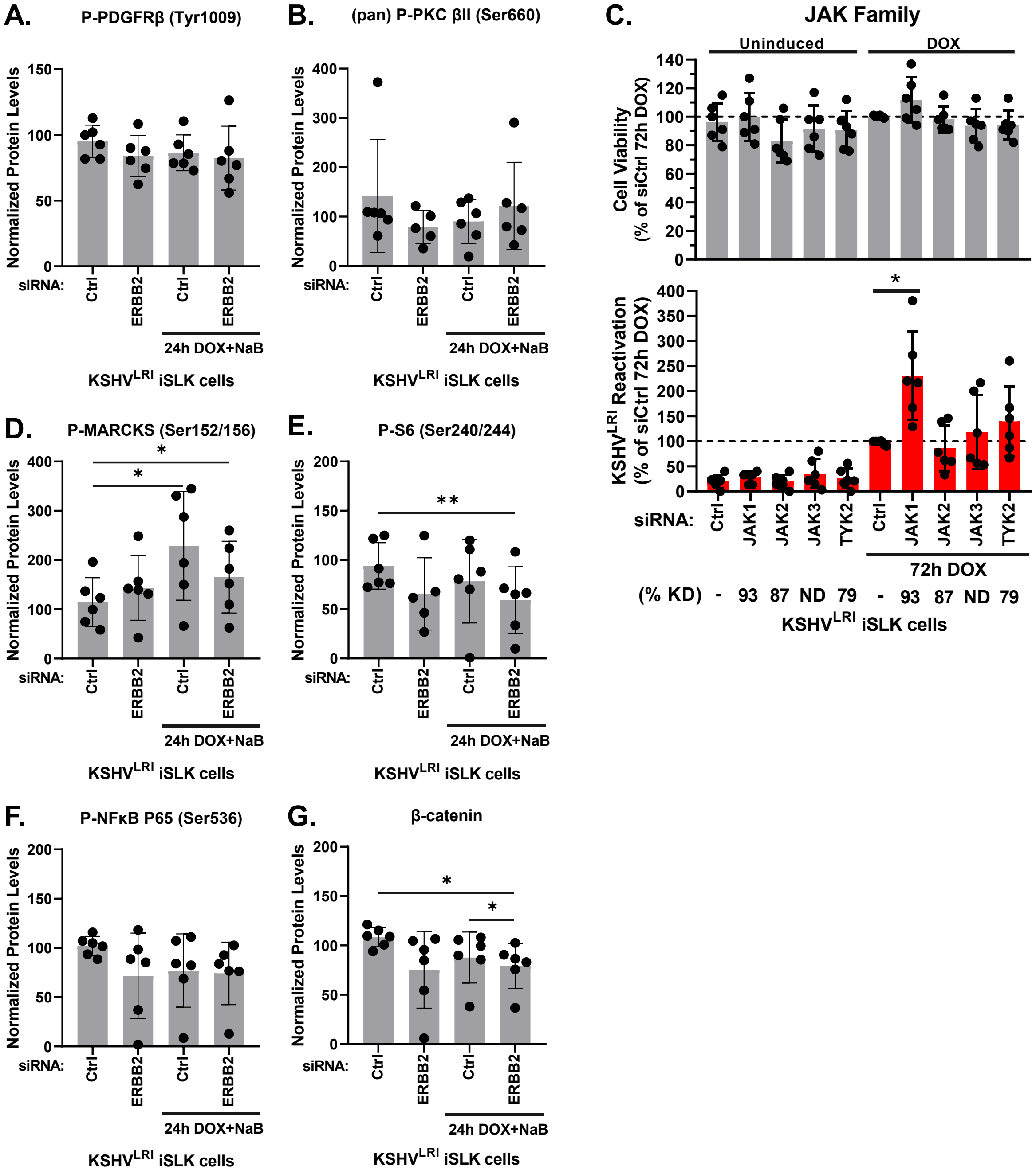

A.
P-PDGFRβ (Tyr1009)
Normalized Protein Levels
siRNA:
Ctrl
ERBB2
24h DOX+NaB
KSHVLRI iSLK cells
B.
(pan) P-PKC βII (Ser660)
C.
JAK Family
Uninduced
DOX
Cell Viability (% of siCtrl 72h DOX)
KSHVLRI Reactivation (% of siCtrl 72h DOX)
JAK1
JAK2
JAK3
TYK2
72h DOX
(% KD)
- 93 87 ND 79
- 93 87 ND 79
D.
P-MARCKS (Ser152/156)
E.
P-S6 (Ser240/244)
F.
P-NFκB P65 (Ser536)
G.
β-catenin

Figure S6

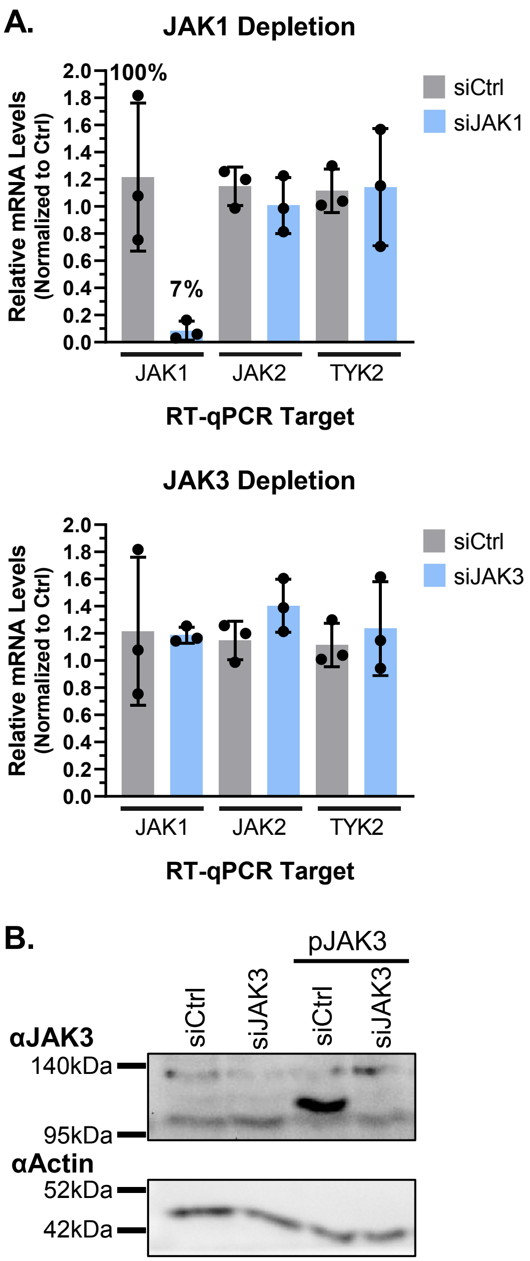

A.
JAK1 Depletion
JAK2 Depletion
JAK3 Depletion
TYK2 Depletion
Relative mRNA Levels (Normalized to Ctrl)
RT-qPCR Target
JAK1
JAK2
TYK2
siCtrl
siJAK1
siJAK2
siJAK3
siTYK2
100%
7%
13%
21%
B.
pJAK3
siCtrl
siJAK3
αJAK3
140kDa
95kDa
αActin
52kDa
42kDa

S1 Table. Kinase inhibitors list

Kinase Inhibitor	Rank	Experiment	CAS #	Consortium / Vender	Catalog ID
Baricitinib	-	Initial screen	1187594-09-7	NCATS	-
Doramapimod	-	Initial screen	285983-48-4	NCATS	-
JNK Inhibitor VIII	-	Initial screen	894804-07-0	NCATS	-
VX-11e	-	Initial screen	896720-20-0	NCATS	-
Rho Kinase Inhibitor III	-	Initial screen	7272-84-6	NCATS	-
PP121	-	Initial screen	1092788-83-4	NCATS	-
GZD824 Dimesylate	-	Initial screen	1421783-64-3	NCATS	-
Staurosporine	-	Initial screen	62996-74-1	NCATS	-
AZD3463	-	Initial screen	1356962-20-3	NCATS	-
NVP-BVU972	-	Initial screen	1185763-69-2	NCATS	-
Y-33075 Dihydrochloride	-	Initial screen	173897-44-4	NCATS	-
Cobimetinib	-	Initial screen	934660-93-2	NCATS	-
WZ3146	-	Initial screen	1214265-56-1	NCATS	-
Aminopurvalanol A	-	Initial screen	220792-57-4	NCATS	-
SB 202190	-	Initial screen	152121-30-7	NCATS	-
PD 166285	-	Initial screen	212391-63-4	NCATS	-
PRT062607	-	Initial screen	1370261-96-3	NCATS	-
Akt Inhibitor VIII	-	Initial screen	612847-09-3	NCATS	-
CP-547632	-	Initial screen	252003-65-9	NCATS	-
Bosutinib	-	Initial screen	380843-75-4	NCATS	-
AT7867	-	Initial screen	857531-00-1	NCATS	-
Cdk1/2 Inhibitor III	-	Initial screen	443798-55-8	NCATS	-
PDK1/Akt/Flt Dual Pathway Inhibitor	-	Initial screen	331253-86-2	NCATS	-
SB 218078	-	Initial screen	135897-06-2	NCATS	-
SCHEMBL2227980	-	Initial screen	1221153-14-5	NCATS	-
Ruboxistaurin Mesylate	-	Initial screen	192050-59-2	NCATS	-
Cot Inhibitor-2	-	Initial screen	915363-56-3	NCATS	-
N/A	-	Initial screen	185039-99-0	NCATS	-
Nintedanib	-	Initial screen	928326-83-4	NCATS	-
PF-477736	2	Validation Set	952021-60-2	Sigma	PZ0186
AZD3463	3	Validation Set	1356962-20-3	Sigma	SML2590
GSK-650394	4	Validation Set	890842-28-1	MedChemExpress	HY-15192

AV 412	5	Validation Set	451492-95-8	MedChemExpress	HY-10346A
ASP-3026	6	Validation Set	1097917-15-1	MedChemExpress	HY-13326
Gefitinib	7	Validation Set	184475-35-2	Sigma	SML1657
Lestaurtinib	8	Validation Set	111358-88-4	Sigma	C7869
Afatinib	9	Validation Set	850140-72-6	MedChemExpress	HY-10261
K252a	14	Validation Set	99533-80-9	Abcam	AB120419
PKR Inhibitor	18	Validation Set	608512-97-6	Calbiochem/Sigma	527451
IRAK1/4 Inhibitor	140	Validation Set	509093-47-4	Sigma	I5409
JAK3 Inhibitor VI	205	Validation Set	856436-16-3	Sigma	420126
Tofacitinib	-	JAK Family	540737-29-9	LC Laboratories	T-1377

S2 Table. DOX plus NaB condition KiR screen final predicted kinase inhibitors

Kinase Inhibitor	Predicted KI response at 0.5µM
Lestaurtinib 0.5uM	0.171
K252a 0.5uM	0.232
Staurosporine 0.5uM	0.241
AP26113 0.5uM	0.251
AZD3463 0.5uM	0.281
WZ8040 0.5uM	0.388
NCGC00344999 0.5uM	0.391
WZ4002 0.5uM	0.408
Go6976 0.5uM	0.409
SB 218078 0.5uM	0.433
WZ3146 0.5uM	0.435
Go6976 0.05uM	0.441
GSK-650394 0.5uM	0.447
A-770041 0.5uM	0.466
AV 412 0.5uM	0.484
SU11652 0.5uM	0.503
NVP-TAE684 0.5uM	0.506
Src Kinase Inhibitor I 0.5uM	0.513
NCGC00263020-01 0.5uM	0.536
PF-3644022 0.5uM	0.540
Milciclib (PHA-848125) 0.5uM	0.546
Indirubin Derivative E804 0.5uM	0.553
JAK3 Inhibitor VI 0.5uM	0.554
Dorsomorphin hydrochloride 0.5uM	0.576
Lck Inhibitor (RK 24466) 0.5uM	0.577
Pluripotin 0.5uM	0.577
EGFR/ErbB2/ErbB4 Inhibitor 0.5uM	0.577
Y-39983 dihydrochloride 0.5uM	0.592
LY2835219 0.5uM	0.595
Dorsomorphin (BML-275, Compound C) 0.5uM	0.605
PDK1/Akt/Flt Dual Pathway Inhibitor 0.5uM	0.608
Neratinib 0.5uM	0.612

MGCD-265 0.5uM	0.617
NVP-TAE226 0.5uM	0.623
MK-5108 (VX-689) 0.5uM	0.623
BMS-536924 0.5uM	0.624
Masitinib 2uM	0.629
GZD824 0.5uM	0.629
BAG 956 0.5uM	0.632
IRAK1/4 Inhibitor 2uM	0.635
XL647 0.5uM	0.640
Cot inhibitor-2 0.5uM	0.642
GSK-3 Inhibitor IX 0.5uM	0.642
Rho Kinase Inhibitor III Rockout 0.5uM	0.653
WHI-P154 0.5uM	0.657
Pim-1 Inhibitor 2 0.5uM	0.661
Cobimetinib (GDC-0973, RG7420) 0.5uM	0.663
LDN-193189 0.5uM	0.664
ASP-3026 0.5uM	0.669
AC480 (BMS-599626) 0.5uM	0.676
LDK378 0.5uM	0.677
GSK1838705A 0.5uM	0.681
Sunitinib (free base) 0.5uM	0.687
Dasatinib 0.5uM	0.691
NCGC00348110 0.5uM	0.691
PKC412 (CGP41251, Midostaurin) 0.5uM	0.697
CC-401 0.5uM	0.700
PDGFR Tyrosine Kinase Inhibitor V 0.5uM	0.700
GSK-3 Inhibitor X 0.5uM	0.703
Foretinib (GSK1363089) 0.5uM	0.707
Gefitinib 2uM	0.708
Compound 56 0.5uM	0.709
KW-2449 0.5uM	0.711
Afatinib (BIBW2992) 0.5uM	0.713
EGFR/ErbB2 Inhibitor 0.5uM	0.716
Allitinib (AST1306) 0.5uM	0.722

AG 1478 0.5uM	0.725
Cdk4 Inhibitor 0.5uM	0.726
Ponatinib (AP24534) 0.5uM	0.727
MLN2480 (BIIB-024, TAK-580) 0.5uM	0.734
CHIR-124 (SB19469) 0.5uM	0.737
GSK-3 Inhibitor XIII 0.5uM	0.740
IRAK inhibitor 5 0.5uM	0.741
BPIQ-I 0.5uM	0.742
TG003 0.5uM	0.743
AMG-51 0.5uM	0.744
CP 724,714 0.5uM	0.748
GSK-3b Inhibitor XII (TWS119) 0.5uM	0.751
JNJ-28312141 0.5uM	0.752
Masitinib 0.5uM	0.752
AMG-25 0.5uM	0.754
Gefitinib 0.5uM	0.755
Bosutinib 0.05uM	0.755
Saracatinib (AZD0530) 0.5uM	0.756
AZD8931 (Sapitinib) 0.5uM	0.762
BGT226 (NVP-BGT226) 0.5uM	0.767
CX-4945 0.5uM	0.767
CP-673,451 0.5uM	0.767
JAK Inhibitor I 0.5uM	0.769
Isogranulatimide 0.5uM	0.770
PKR Inhibitor (Negative Control) 0.5uM	0.776
GTP-14564 0.5uM	0.778
PI3Kg Inhibitor (AS-605240) 2uM	0.783
Go 6983 0.5uM	0.788
PD 166285 dihydrochloride 0.5uM	0.789
AT9283 0.5uM	0.791
GSK-3b Inhibitor XII (TWS119) 0.05uM	0.791
Erlotinib HCl 2uM	0.791
Indirubin-3-monoxime 0.5uM	0.793
Emodin (Archin, Frangulic Acid) 0.5uM	0.793

Flt3 Inhibitor III 0.5uM	0.794
Syk Inhibitor (OXSI 2) 0.5uM	0.794
PD 174265 0.5uM	0.796
NVP-BEZ235 (Dactolisib) 0.5uM	0.797
AMG-208 0.5uM	0.799
Lapatinib 0.5uM	0.800
IRAK1/4 Inhibitor 0.5uM	0.800
c-Met/RON Dual Kinase Inhibitor 0.5uM	0.801
AG-1296 0.5uM	0.801
Takeda-6d 0.5uM	0.804
Baricitinib (LY3009104, INCB028050) 0.5uM	0.804
VEGFR2 Kinase Inhibitor I 0.5uM	0.804
Pazopanib 0.5uM	0.804
TGFb RI Kinase Inhibitor (LY364947, HTS 466284) 0.5uM	0.805
VEGFR Tyrosine Kinase Inhibitor II 0.5uM	0.805
VEGFR2 Kinase Inhibitor IV 0.5uM	0.806
Flt3 Inhibitor II 0.5uM	0.806
PI-103 5uM	0.807
PP121 0.5uM	0.809
S-99 0.5uM	0.809
ROCK Inhibitor Y-27632 0.5uM	0.811
STO-609 0.5uM	0.814
Vemurafenib (PLX4032, RG7204) 0.5uM	0.815
Syk Inhibitor II hydrochloride 0.5uM	0.817
GSK690693 0.5uM	0.819
SKF-86002 0.5uM	0.820
PD 158780 0.5uM	0.820
Crenolanib (CP-868596) 0.5uM	0.823
VEGFR2 Kinase Inhibitor II 0.5uM	0.823
Vargatef (Nintedanib, BIBF 1120) 0.5uM	0.823
SU6656 0.5uM	0.825
KU-55933 (ATM Kinase Inhibitor) 5uM	0.825
PDGFR Tyrosine Kinase Inhibitor IV 0.5uM	0.825
GSK-3b Inhibitor XI 0.5uM	0.826

PD 169316 0.5uM	0.827
Cdk4 Inhibitor III (Ryuvidine) 0.5uM	0.828
Cdk1/2 Inhibitor III 0.5uM	0.828
Danusertib (PHA-739358) 0.5uM	0.828
SKI II 0.5uM	0.828
Tandutinib 2uM	0.828
HA-1077 Dihydrochloride (Fasudil) 0.5uM	0.829
SB203580 0.5uM	0.830
PD98059 5uM	0.831
AR-A014418 0.5uM	0.831
BIIB-057 (PRT062607, P505-15) 0.5uM	0.831
Bohemine 0.5uM	0.832
ERK Inhibitor III 0.5uM	0.833
Cdk1/5 Inhibitor (NSC 693868) 0.5uM	0.835
JNK Inhibitor V 0.5uM	0.835
NVP-ADW742 0.5uM	0.836
Palbociclib (PD-0332991) 0.5uM	0.837
SC-68376 0.5uM	0.838
AMG-458 0.5uM	0.840
Akt-I-1,2 0.5uM	0.840
MK-8776 (SCH 900776) 0.5uM	0.841
AG 112 (Tyrphostin A48) 0.5uM	0.841
Tivozanib (AV-951) 0.5uM	0.842
Erlotinib HCl 0.5uM	0.843
GSK-3b Inhibitor II 0.5uM	0.844
Chelerythrine Chloride 0.5uM	0.844
Rho Kinase Inhibitor IV 0.5uM	0.844
SB202190 (FHPI) 0.5uM	0.845
PKCbII/EGFR Inhibitor 0.5uM	0.846
Chk2 Inhibitor II 2uM	0.847
Cdk4 Inhibitor II (NSC 625987) 0.5uM	0.847
Triciribine 5uM	0.847
GSK-269962A 0.5uM	0.848
Rapamycin (Sirolimus) 0.5uM	0.848

Akt Inhibitor IV 0.5uM	0.850
Chk2 Inhibitor II 0.5uM	0.850
GW441756 0.5uM	0.851
SB 202474 0.5uM	0.851
Purvalanol A 0.5uM	0.851
Fascaplysin Synthetic 0.5uM	0.852
SB220025 0.5uM	0.852
NVP-BVU972 0.5uM	0.853
JNK Inhibitor II (SP6) 0.5uM	0.853
KRN 633 2uM	0.856
p38 MAP Kinase Inhibitor 0.5uM	0.856
Alsterpaullone 2 Cyanoethyl 0.5uM	0.857
BMS-794833 0.5uM	0.858
NCGC00350264-01 0.5uM	0.859
PD173955 0.5uM	0.860
Sunitinib malate (SU11248) 0.5uM	0.861
Sorafenib 0.5uM	0.862
KRN 633 0.5uM	0.863
Tie2 kinase inhibitor 0.5uM	0.864
AG 9 (Tyrphostin A1) 0.5uM	0.865
SL-327 5uM	0.865
Cediranib (AZD2171) 0.5uM	0.865
GNF-2 0.5uM	0.867
MNS (3,4-Methylenedioxy-B-nitrostyrene, MDBN) 0.5uM	0.867
JNJ-7706621 0.05uM	0.867
GSK-1070916 0.5uM	0.867
PHT-427 0.5uM	0.869
Dovitinib (TKI-258, CHIR-258) 0.5uM	0.869
GSK-3 Inhibitor IX 0.05uM	0.869
Wortmannin 0.5uM	0.870
Brivanib (BMS-540215) 0.5uM	0.871
SB202190 (FHPI) 2uM	0.872
MK-2461 0.5uM	0.872
PD98059 0.5uM	0.872

PF-04691502 0.5uM	0.873
DNA-PK Inhibitor III 0.5uM	0.873
GDC-0941 0.5uM	0.873
Diacylglycerol Kinase Inhibitor II 0.5uM	0.873
Alsterpaullone 0.5uM	0.873
JAK Inhibitor I 0.05uM	0.874
SR-3306 0.5uM	0.874
H-89 Dihydrochloride 0.5uM	0.874
AG-1024 2uM	0.874
AG-490 (Tyrphostin B42) 0.5uM	0.875
Pazopanib HCl (GW786034 HCl) 0.5uM	0.875
Imatinib (Gleevec) 2uM	0.875
PD 173955 Analogue 1 0.5uM	0.876
Nilotinib (AMN-107) 0.5uM	0.876
VEGFR2 Kinase Inhibitor III 0.5uM	0.877
KU-55933 (ATM Kinase Inhibitor) 0.5uM	0.878
QNZ (EVP4593) 0.5uM	0.878
Cdk1 Inhibitor 0.5uM	0.878
Imatinib Mesylate 0.5uM	0.879
Sorafenib Tosylate 0.5uM	0.879
PI-103 0.5uM	0.879
Aloisine A (RP107) 0.5uM	0.879
AG-490 (Tyrphostin B42) 5uM	0.880
Linifanib (ABT-869) 0.5uM	0.882
SB590885 0.5uM	0.882
EGFR Inhibitor 0.5uM	0.883
Casein Kinase II Inhibitor III (TBCA) 0.5uM	0.883
CAL-101 (Idelalisib, GS-1101) 0.5uM	0.883
PP1 Analog II 1NM-PP1 0.5uM	0.884
Cabozantinib malate (XL184) 0.5uM	0.884
PDGFR Tyrosine Kinase Inhibitor II 0.5uM	0.884
Akt Inhibitor VIII Isozyme Selective Akti-1/2 0.5uM	0.884
GSK-3b Inhibitor I (TDZD-8) 0.5uM	0.885
Copanlisib (BAY 80-6946) 0.5uM	0.885

KX2-391 0.5uM	0.885
SKI II 5uM	0.886
Tpl2 Kinase Inhibitor 0.5uM	0.886
OSI-930 0.5uM	0.886
NVP-AEW541 0.5uM	0.887
PDGFR Tyrosine Kinase Inhibitor III 0.5uM	0.887
CGI1746 0.5uM	0.887
Piceatannol 0.5uM	0.887
IMD 0354 0.5uM	0.888
AZ 23 0.5uM	0.888
ITD 1 0.5uM	0.889
Apatinib 0.5uM	0.889
LY2157299 0.5uM	0.889
BMS-2 (Met/Flt-3/VEGFR2 TKI) 0.5uM	0.889
MLN120B 0.5uM	0.890
Cdk2 Inhibitor IV (NU6140) 0.5uM	0.890
PRP003-073 0.5uM	0.890
Arry-380 0.5uM	0.891
DNA-PK Inhibitor II 0.5uM	0.891
BGJ398 (Infigratinib) 0.5uM	0.892
Cdk/Crk Inhibitor (RGB-286147, Cdk7 Inhibitor IV) 0.5uM	0.892
Casein Kinase I Inhibitor (D4476) 0.5uM	0.892
SCIO-469 (Talmapimod HCl) 0.5uM	0.892
Triciribine 0.5uM	0.893
DNA-PK Inhibitor V 0.5uM	0.893
Wortmannin 2uM	0.893
Bentamapimod (AS-602801) 0.5uM	0.894
AGL 2043 0.5uM	0.894
Mubritinib (TAK 165) 0.5uM	0.894
BKM120 (NVP-BKM120, Buparlisib) 0.5uM	0.895
INCB 28060 (Capmatinib) 0.5uM	0.895
Bosutinib 0.5uM	0.895
KN-93 5uM	0.895
Tofacitinib 2uM	0.896

AG-1024 0.5uM	0.896
BYL719 0.5uM	0.897
CGK 733 0.5uM	0.897
RAF265 (CHIR-265) 0.5uM	0.898
ZSTK474 0.5uM	0.898
BI 78D3 0.5uM	0.900
GSK1904529A 0.5uM	0.900
PRT-060318 0.5uM	0.900
Akt Inhibitor VIII Isozyme Selective Akti-1/2 5uM	0.900
OSI-906 (Linsitinib) 0.5uM	0.901
p38 MAP Kinase Inhibitor III 0.5uM	0.902
JNK Inhibitor (Negative Control) 0.5uM	0.902
TGFb RI Inhibitor III 0.5uM	0.903
GNF-2 5uM	0.903
Herbimycin A 0.5uM	0.904
Motesanib Diphosphate 0.5uM	0.904
Cdk2 Inhibitor III (CVT-313) 0.5uM	0.905
PI3Kg Inhibitor (AS-605240) 0.5uM	0.905
Tandutinib 0.5uM	0.905
AZD5363 0.5uM	0.905
SGX-523 0.5uM	0.905
ZM 336372 0.5uM	0.905
AS-604850 5uM	0.906
JAK3 Inhibitor IV 0.5uM	0.906
GF109203X 0.5uM	0.906
AKT-I-1 0.5uM	0.908
Telatinib 0.5uM	0.908
KN-93 0.5uM	0.908
SL-327 0.5uM	0.909
Roscovitine (Seliciclib, CYC202) 2uM	0.910
Aloisine (RP106) 0.5uM	0.910
GDC-0068 0.5uM	0.910
BI-2536 0.5uM	0.910
BAY 11-7082 0.5uM	0.910

Bisindolylmaleimide IV 0.5uM	0.910
PKCb Inhibitor 0.5uM	0.910
Casein Kinase I Inhibitor (D4476) 5uM	0.910
c-FMS inhibitor 0.5uM	0.911
SB505124 0.5uM	0.911
AZD8055 0.5uM	0.911
GSK-3 INHIBITOR-I 0.5uM	0.911
SAR245409 (XL765) 0.5uM	0.912
IC261 0.5uM	0.913
TSU-68 (SU6668, Orantinib) 0.5uM	0.913
GSK2334470 0.5uM	0.914
Kenpaullone 0.5uM	0.914
JNJ-38877605 0.5uM	0.914
KU-60019 0.5uM	0.915
PF-05212384 (PKI-587) 0.5uM	0.915
FLT3 Inhibitor (TCS 359) 0.5uM	0.915
Control	0.915
MEK Inhibitor I 0.5uM	0.915
PHA-690509 0.5uM	0.916
OSU-03012 (AR-12) 0.5uM	0.918
Ku-0060648 0.5uM	0.918
PKC412 (CGP41251, Midostaurin) 0.05uM	0.918
Roscovitine (Seliciclib, CYC202) 0.5uM	0.919
PQ 401 0.5uM	0.919
FR 180204 0.5uM	0.920
Rapamycin (Sirolimus) 5uM	0.920
VE-821 (ATR Inhibitor IV) 0.5uM	0.920
CGP 57380 0.5uM	0.920
Endoxifen 0.5uM	0.920
Aminopurvalanol A 0.5uM	0.921
LY 294002 5uM	0.921
PKR Inhibitor 0.5uM	0.921
AMG-Tie2-1 0.5uM	0.922
XL147 0.5uM	0.922

IC-87114 0.5uM	0.922
SB203580 2uM	0.922
Fingolimod (FTY720) HCl 0.5uM	0.923
Dovitinib (TKI-258, CHIR-258) 0.05uM	0.923
KN-62 0.5uM	0.923
VX-702 5uM	0.923
GDC-0980 (RG7422) 0.5uM	0.924
BMS-5 (LIM Kinase Inhibitor I) 0.5uM	0.924
PF-4708671 0.5uM	0.925
PF-3758309 0.5uM	0.925
MK2a Inhibitor 0.5uM	0.926
TG101348 (SAR302503) 0.5uM	0.927
RWJ 67657 (JNJ-3026582) 0.5uM	0.928
Akt Inhibitor X 0.5uM	0.928
AZD-26 0.5uM	0.929
Dinaciclib (SCH727965) 0.5uM	0.930
Mubritinib (TAK 165) 5uM	0.932
Vandetanib 0.5uM	0.932
Amuvatinib (MP-470) 0.5uM	0.932
Tofacitinib 0.5uM	0.932
Axitinib 0.5uM	0.933
GNF-5837 0.5uM	0.933
GSK2126458 (GSK458) 0.5uM	0.934
Hydroxocobalamin (Vitamin B12a) 0.5uM	0.934
MEK Inhibitor II 0.5uM	0.934
Aurora A Inhibitor I 0.5uM	0.934
AG-1295 0.5uM	0.937
Compound 52 (NG 52) 0.5uM	0.937
MK-22-6 0.5uM	0.938
AZD7545 0.5uM	0.938
JNK Inhibitor VIII 0.5uM	0.938
Tepotinib (EMD 1214063) 0.5uM	0.942
S6K-18 0.5uM	0.944
SU9516 0.5uM	0.944

Doramapimod (BIRB 796, p38 MAP Kinase Inhibitor X) 0.5uM	0.944
AS-604850 0.5uM	0.945
Regorafenib (BAY 73-4506) 0.5uM	0.945
Rho Kinase Inhibitor IV 2uM	0.951
Vatalanib (PTK787) 2HCl 0.5uM	0.952
Quizartinib HCl (AC-220) 0.5uM	0.953
Flavopiridol (Alvocidib) 0.5uM	0.953
7307-50-7 0.5uM	0.955
JNK Inhibitor IX 5uM	0.955
NCGC00371288-02 0.5uM	0.955
Indirubin Derivative E804 0.05uM	0.956
BMS-3 0.5uM	0.957
NCGC00244250-01 0.5uM	0.957
PF-477736 0.5uM	0.958
LY 333531 mesylate (Ruboxistaurin) 0.5uM	0.959
GW2580 0.5uM	0.959
SU 4312 (DMBI) 0.5uM	0.960
Cdk1 Inhibitor (CGP74514A) 0.5uM	0.962
MK-2206 2HCl 0.5uM	0.964
AT7867 0.5uM	0.966
Aurora Kinase Inhibitor III 0.5uM	0.967
Filgotinib (GLPG0634) 0.5uM	0.968
JNK Inhibitor IX 0.5uM	0.969
LY2603618 0.5uM	0.970
FR 180204 2uM	0.971
Idronoxil (Phenoxodiol) 0.5uM	0.972
PP3 0.5uM	0.972
CP 547,632 0.5uM	0.973
SB-747651A 0.5uM	0.976
VX-702 0.5uM	0.979
NVP-231 0.5uM	0.981
LY 303511 (Negative control) 0.5uM	0.983
LY 294002 0.5uM	0.986
SGI-1776 free base 0.5uM	0.987

CEP-33779 0.5uM	0.987
PF-00562271 0.5uM	0.988
MK-1775 0.5uM	0.992
JNJ-7706621 0.5uM	0.994
Met Kinase Inhibitor (SU11274) 0.5uM	0.998
Enzastaurin (LY317615) 0.5uM	1.002
TPCA-1 0.5uM	1.004
FMK (RSK inhibitor Fmk) 0.5uM	1.024
CHEMBL2336015 0.5uM	1.025
BI-D1870 0.5uM	1.030
PF-573228 0.5uM	1.030
Ro-32-0432 hydrochloride 0.5uM	1.032
MLN8054 0.5uM	1.036
Sotrastaurin (AEB071) 0.5uM	1.040
Alisertib (MLN8237) 0.5uM	1.043
BX-912 0.5uM	1.053
RO-495 0.5uM	1.069
VX-680 (Tozasertib, MK-0457) 0.5uM	1.086
Crizotinib (PF-02341066) 0.5uM	1.121
LY2784544 0.5uM	1.121
TCS ERK 11e (ERK Inhibitor VIII, VX-11e) 0.5uM	1.144
BMS-754807 0.5uM	1.157

S3 Table. DOX plus NaB condition KiR screen final predicted kinases

Number	Kinase	Average Coefficient
1	ITK	0.00247
2	*PKG2 (PRKG2)**	0.00223
3	MAP3K8 (COT1)	0.00210
4	FRK	0.00183
5	CLK1	0.00191
6	ERBB4 (HER4)	0.00168
7	MAP2K2 (MEK2)	0.00125
8	CAMK2G	0.00083
9	MKNK2 (MNK2)	0.00086
10	PBK	0.00079
11	TEC	0.00072
12	LRRK2	0.00055
13	MAP4K4 (HGK)	0.00045
14	DSTYK (RIPK5)	0.00066

** No expression data for iSLK cells containing latent or lytic replicating virus [34]. Excluded from validated kinases.*

S4 Table. Kinase expression data from KSHV BAC16 infected iSLK cells

Gene	Description	Latent	Induced (48h DOX+NaB)
CAMK2G	calcium/calmodulin dependent protein kinase II gamma	17.8	19.3
CLK1	Cell-division contol like kinase 1	11.2	13.1
DSTYK (RIPK5)	dual serine/threonine and tyrosine protein kinase	5.1	3.0
ERBB1	epidermal growth factor receptor	72.7	41.6
ERBB2	erb-b2 receptor tyrosine kinase 2	16.6	12.8
ERBB3	erb-b2 receptor tyrosine kinase 3	0.9	7.4
ERBB4	erb-b2 receptor tyrosine kinase 4	0.000	0.049
FRK	fyn related Src family tyrosine kinase	0.9	6.6
ITK	IL2 inducible T-cell kinase	0.06	0.55
LRRK2	leucine rich repeat kinase 2	0.000	0.008
MAP2K2 (MEK2)	mitogen-activated protein kinase kinase 2	63.3	86.0
MAP3K8 (COT1)	mitogen-activated protein kinase kinase kinase 8	1.2	3.2
MAP4K4 (HGK)	mitogen-activated protein kinase kinase kinase kinase 4	19.6	28.2
MKNK1 (MNK1)	MAP kinase interacting serine/threonine kinase 1	11.0	8.1
MKNK2 (MNK2)	MAP kinase interacting serine/threonine kinase 2	20.6	56.0
PBK	PDZ binding kinase	42.6	11.4
TEC	tec protein tyrosine kinase	1.9	1.3

From published RNA-seq dataset GSE157275 [34]

S5 Table. Primers

Primer Name	Primer #	Oligo Sequence (5' - 3')	Assay	Annealing Tm	Reference
HindIII PAN promoter Fwd	2580	GGA GAA GCT TTA TGG AGT TTT CTT ATG GAT TAT TAA GGG TCA GCT TGA AGG	PCR	65°C	This paper
PAN promoter HindIII Rev	2581	CCT AAA GCT TCC AAG GTG ACT GGG CAG TCC CAG TGC TAA ACT GAC TCA A	PCR	65°C	This paper
KpnI-SP-SBPdeltaLRNGR Fwd	2585	CTT AGG TAC CAT GGG ATG GTC CTG CAT CAT CC	PCR	61°C	This paper
BamHI-deltaLNGFR Rev	2586	GAA TGG ATC CAG CGC GCT TGA AGG CAA TAT AGG CC	PCR	61°C	This paper
BamHI-P2A-mCherry Fwd	2600	GTT AGG ATC CGG CGC AAC AAA CTT CTC TCT GCT GAA ACA AGC CGG AGA TGT CGA AGA GAA TCC TGG ACC GAT GGT GAG CAA GGG CGA GG	PCR	65°C	This paper
NotI-NLS-mCherry Rev	2601	CAT TGC GGC CGC TTA TTG GAC CTT CCG CTT TTT CTT CG	PCR	65°C	This paper
BamHI-50bpREPSEQ-SceI-KanR Fwd	2610	GTT AGG ATC CGG CGC AAC AAA CTT CTC TCT GCT GAA ACA AGC CGG AGA TGT CGA AGA GAA TAG GGA TAA CAG GGT AAT CGA TTT	PCR	65°C	This paper
BamHI-KanR Rev	2611	CTG CCT GAC CTT GGA TCC GCC AGT GTT ACA ACC AAT TAA CC	PCR	65°C	This paper
61bp-KSHV-LNGFR-mCherry Cassette	2617	TCG GCA GAA TGC TTA ATG AAT TAC AAC AGT ACT GCG ATG AGT GGC AGG GCG GGG CGT AAT TTA TGG AGT TTT CTT ATG GAT TAT TAA GGG	PCR	63°C	This paper
70bp-KSHV-LNGFR-mCherry Cassette	2618	CGC TTA TTA TCA CTT ATT CAG GCG TAG CAA CCA GGC GTT TAA GGG CAC CAA TAA CTG CCT TAA AAA AAT TCC ATA GAG CCC ACC GCA TCC	PCR	63°C	This paper
Kozak-JAK3 Fwd	2636	CGC CAC CAT GGC ACC TCC AAG TGA AGA G	PCR; Sanger seq.	59°C; 60°C	This paper
JAK3_2 Rev	2648	CGA CTC ACT ATA GGG GAT ATC AGC TGG ATG G	PCR	59°C	This paper
mid-JAK3 Rev	2638	GCA CAG GTC CTC AGC CAA GTG G	Sanger seq.	60°C	This paper
mid-JAK3_1 Fwd	2646	GCT GCC GCT TGA CAA AGA CTA CTA	Sanger seq.	60°C	This paper
CAMK2G F qPCR	2773	ACC CGT TTC ACC GAC GAC TA	Quantitative PCR	60°C	PMID: 35039634
CAMK2G R qPCR	2774	CTC CTG CGT GGA GGT TTT CTT	Quantitative PCR	60°C	PMID: 35039634
CLK1 F qPCR	2753	TGA ATA CTA TCT TGG GTT TAC CGT AT	Quantitative PCR	55°C	PMID: 29606096

CLK1 R qPCR	2754	CGT TTC CTG GTT TTC TGT ATC ATA T	Quantitative PCR	55°C	PMID: 29606096
COT1_MAP3K8 F qPCR	2767	GAG CGT TCT AAG TCT CTG CTG	Quantitative PCR	60°C	PMID: 30463908
COT1_MAP3K8 R qPCR	2768	GCA AGC AAA TCC TCC ACA GTT C	Quantitative PCR	60°C	PMID: 30463908
EGFR F qPCR	2757	GGG GC C G AC AGC TA T GAG AT	Quantitative PCR	60°C	PMID: 32978523
EGFR R qPCR	2758	ACC TAT TCC GTT ACA CAC TTTGC	Quantitative PCR	60°C	PMID: 32978523
ERBB2 F qPCR	2759	GCC GTG CTA GAC AAT GGA GA	Quantitative PCR	60°C	PMID: 32978523
ERBB2 R qPCR	2760	TCA AGA TCT CTG TGA GGC TTC G	Quantitative PCR	60°C	PMID: 32978523
ERBB3 F qPCR	2761	ACA TCG TGA GGG ACC GAG AT	Quantitative PCR	60°C	PMID: 32978523
ERBB3 R qPCR	2762	GGT CTT GGT CAA TGT CTG GCA	Quantitative PCR	60°C	PMID: 32978523
ERBB4 F qPCR	2763	ATG GCT TAC AGG GGG CAA AC	Quantitative PCR	60°C	PMID: 32978523
ERBB4 R qPCR	2764	AGT GGG ACC GTT ACA CCC TT	Quantitative PCR	60°C	PMID: 32978523
FRK_PTK5 F qPCR	2755	CTC TGG GAG TAC CTA GAA CCC	Quantitative PCR	60°C	PMID: 31043790
FRK_PTK5 R qPCR	2756	AGC CTG GTA ATC AAA CAA AGC C	Quantitative PCR	60°C	PMID: 31043790
HGK_MAP4K4 F qPCR	2783	GGG GAA CGC TTC AGA GTG AG	Quantitative PCR	60°C	PMID: 26688060
HGK_MAP4K4 R qPCR	2784	GTG CGG TCA GAT CAG CAG G	Quantitative PCR	60°C	PMID: 26688060
ITK F qPCR	2765	ACT CCT GAA GAC AAC AGG CGA	Quantitative PCR	60°C	PMID: 33283362
ITK R qPCR	2766	ATC CTT CAT GCC CAT TCC TGTC	Quantitative PCR	60°C	PMID: 33283362
JAK1 F qPCR	2739	GGG AAA TCT GCT ACA ATG GC	Quantitative PCR	55°C	PMID: 27350337
JAK1 R qPCR	2740	TGA TGG CTC GGA AGA AAG GC	Quantitative PCR	55°C	PMID: 27350337
JAK2 F qPCR	2741	CCA GAT GGA AAC TGT TCG CTC AG	Quantitative PCR	60°C	ORIGENE
JAK2 R qPCR	2742	GAG GTT GGT ACA TCA GAA ACA CC	Quantitative PCR	60°C	ORIGENE
JAK3 F qPCR	2743	GCC TGG AGT GGC ATG AGA A	Quantitative PCR	60°C	PMID: 20673263
JAK3 R qPCR	2744	CCC CGG TAA ATC TTG GTG AA	Quantitative PCR	60°C	PMID: 20673263
K8.1 F qPCR	2680	AAA GCG TCC AGG CCA CCA CAG	Quantitative PCR	60°C	PMID: 28275189
K8.1 R qPCR	2681	GGC AGA AAA TGG CAC ACG GTT	Quantitative PCR	60°C	PMID: 28275189
LRRK2 F qPCR	2781	GGA TGT TGG TGA TGG AGT T	Quantitative PCR	55°C	PMID: 29545945
LRRK2 R qPCR	2782	GGC TGA GTG GAG GTA TCT	Quantitative PCR	55°C	PMID: 29545945
mCherry-ato F qPCR	2791	TTC AGC CTC TGC TTG ATC TC	Quantitative PCR	60°C	PMID: 22216235
mCherry-ato R qPCR	2792	GCG CGT GAT GAA CTT CGA G	Quantitative PCR	60°C	PMID: 22216235
MEK2_MAP2K2 F qPCR	2771	CCA AGG TCG GCG AAC TCA AA	Quantitative PCR	60°C	PMID: 33226073
MEK2_MAP2K2 R qPCR	2772	TCT CAA GGT GGA TCA GCT TCC	Quantitative PCR	60°C	PMID: 33226073
MKNK1 F qPCR	2775	GAG ATG GGC AGT AGC GAA CCC C	Quantitative PCR	60°C	PMID: 31894299
MKNK1 R qPCR	2776	GGC TCA CGG CAC CTT GAA CTT T	Quantitative PCR	60°C	PMID: 31894299
MKNK2 F qPCR	2777	AGG AAG ATG TGC TGG GGG AG	Quantitative PCR	60°C	PMID: 23365451

MKNK2 R qPCR	2778	CCT GGC ACT GGT ACA GCA TC	Quantitative PCR	60°C	PMID: 23365451
ORF10 F qPCR	2678	GTC CTG TCC CGC TCT CTT TTT TG	Quantitative PCR	60°C	PMID: 28275189
ORF10 R qPCR	2679	CAG TAA GGT GTT CGT GCT TGC CC	Quantitative PCR	60°C	PMID: 28275189
PBK_TOPK F qPCR	2779	GAA GAG GAC TGA GAG TGG CT	Quantitative PCR	60°C	PMID: 27049917
PBK_TOPK R qPCR	2780	CTT CTG CAT AAA CGG AGA GGC	Quantitative PCR	60°C	PMID: 27049917
RIPK5_DSTYK F qPCR	2785	TCA GGA AAT CCG AAA GTA	Quantitative PCR	55°C	PMID: 31201369
RIPK5_DSTYK R qPCR	2786	CTC AGA TAG CCC AGG TCA	Quantitative PCR	55°C	PMID: 31201369
TEC F qPCR	2751	AAC TGA AAA ATT AGC ACC CGG A	Quantitative PCR	55°C	PMID: 33516764
TEC R qPCR	2752	ATG GCT ACA ACG ATT TCT TCA CT	Quantitative PCR	55°C	PMID: 33516764
Tubulin F qPCR	2682	TCC AGA TTG GCA ATG CCT G	Quantitative PCR	60°C	PMID: 19158252
Tubulin R qPCR	2683	GGC CAT CGG GCT GGA T	Quantitative PCR	60°C	PMID: 19158252
TYK2 F qPCR	2745	GTG GCA GCA GTG GCA GGA AC	Quantitative PCR	60°C	PMID: 32694928
TYK2 R qPCR	2746	CTC AGC TCC AGG CAC TTG TTG TC	Quantitative PCR	60°C	PMID: 32694928

S6 Table. Dharmacon siRNA target and ID

siRNA Target	Dharmacon Catalog ID
CAMK2G	L-004536-00-0005
CLK1	L-004800-00-0005
DSTYK	L-004049-00-0005
ERBB1	L-003114-00-0005
ERBB2	L-003126-00-0005
ERBB3	L-003127-00-0005
ERBB4	L-003128-00-0005
FRK	L-003139-00-0005
ITK	L-003144-00-0005
JAK1	L-003145-00-0005
JAK2	L-003146-00-0005
JAK3	L-003147-00-0005
LRRK2	L-006323-00-0005
MAP2K2	L-003573-00-0005
MAP3K8	L-003511-00-0005
MAP4K4	L-003971-00-0005
MKNK1	L-004879-00-0005
MKNK2	L-004908-00-0005
Non-targeting control	D-001810-10-05
PBK	L-005390-00-0005
TEC	L-003177-00-0005
TYK2	L-003182-00-0005

S7 Table. Antibodies

Antibody Name	Phospho-site	Vender / Source	Catalog ID / Reference	Assay
Actin	N/A	Sigma	A2066	Immunoblot
β-actin	N/A	Sigma	A1978	Protein microarray
Her2 c-terminus (ERBB2)	N/A	Rabbit derived	Child et al. 1999; PMID: 10216954	Immunoblot
JAK3 (B-12)	N/A	Santa Cruz	sc-6932	Immunoblot
LANA	N/A	Rabbit derived	Lagunoff et al. 2002; PMID: 11836422; a kind gift from A. Polson and D. Ganem	Immunoblot; Immunofluorescence
p75 NGF receptor (LNGFR)	N/A	Abcam	ab52987	Immunoblot
P-AKT	Ser473	Cell Signaling	4058	Protein microarray
P-CREB1	Ser133	Cell Signaling	9198	Protein microarray
P-EGFR (ERBB1)	Tyr1173	Cell Signaling	4407	Protein microarray
P-MARCKS	er152/156	Cell Signaling	2741	Protein microarray
P-MET	Tyr1349	Cell Signaling	3133	Protein microarray
P-NFκB P65	Ser536	Cell Signaling	3033	Protein microarray
P-P44/42 MAPK (ERK1/2)	Thr202/Tyr204	Cell Signaling	4377	Protein microarray
P-PDGFRβ	Tyr1009	Cell Signaling	3124	Protein microarray
P-PKC (pan) βII	Ser660	Cell Signaling	9371	Protein microarray
P-S6 ribosomal protein	Ser240/244	Cell Signaling	2215	Protein microarray
P-SRC	Tyr416	Cell Signaling	2101	Protein microarray
P-STAT1	Tyr701	Cell Signaling	9167	Protein microarray
P-STAT3	Tyr705	Cell Signaling	9145	Protein microarray
Streptavidin, Alexa Fluor 680 conjugate	N/A	Invitrogen	S21378	Immunofluorescence
β-catenin	N/A	Cell Signaling	9582	Protein microarray
Goat αRabbit, Alexa Fluor 488	N/A	Invitrogen	A11008	Immunofluorescence
Goat αRabbit, Alkaline Phosphatase	N/A	Invitrogen	T2191	Immunoblot
Goat αMouse, IRDye 800CW	N/A	LI-COR	926-32210	Protein microarray
Goat αRabbit, IRDye 680LT	N/A	LI-COR	925-68071	Protein microarray
